# Identification of Candidate Synovial Fluid Biomarkers for the Prediction of Patient Outcome After Microfracture or Osteotomy

**DOI:** 10.1177/0363546521995565

**Published:** 2021-03-31

**Authors:** Charlotte H. Hulme, Mandy J. Peffers, Gabriel Mateus Bernardo Harrington, Emma Wilson, Jade Perry, Sally Roberts, Pete Gallacher, Paul Jermin, Karina T. Wright

**Affiliations:** * School of Pharmacy and Bioengineering, Keele University, Keele, Staffordshire, UK; † Robert Jones and Agnes Hunt Orthopaedic Hospital, Oswestry, Shropshire, UK; ‡ Institute of Ageing and Chronic Disease, University of Liverpool, Liverpool, UK; § Centre for Proteome Research, Institute of Integrative Biology, University of Liverpool, Liverpool, UK; ‖ Chester Medical School, Chester University, Chester, UK; Investigation performed at School of Pharmacy and Bioengineering, Keele University, Keele, Staffordshire, UK, and the Robert Jones and Agnes Hunt Orthopaedic Hospital, Oswestry, Shropshire, UK

**Keywords:** early osteoarthritis, knee, microfracture, osteotomy, predictive biomarkers, synovial fluid, proteomics, immunoassays, enzyme activity assays

## Abstract

**Background:**

Biomarkers are needed to predict clinical outcomes for microfracture and osteotomy surgeries to ensure patients can be better stratified to receive the most appropriate treatment.

**Purpose:**

To identify novel biomarker candidates and to investigate the potential of a panel of protein biomarkers for the prediction of clinical outcome after treatment with microfracture or osteotomy.

**Study Design:**

Descriptive laboratory study.

**Methods:**

To identify novel candidate biomarker proteins, we used label-free quantitation after liquid chromatography–tandem mass spectrometry of dynamic range-compressed synovial fluids (SFs) from individuals who responded excellently or poorly (based on change in Lysholm score) to microfracture (n = 6) or osteotomy (n = 7). Biomarkers that were identified in this proteomic analysis or that relate to osteoarthritis (OA) severity or have predictive value in another early OA therapy (autologous cell implantation) were measured in the SF of 19 and 13 patients before microfracture or osteotomy, respectively, using commercial immunoassays, and were normalized to urea. These were aggrecanase-1 (ADAMTS-4), cartilage oligomeric matrix protein (COMP), hyaluronan (HA), lymphatic vessel endothelial hyaluronan receptor 1 (LYVE-1), matrix metalloproteinase 1 and 3, soluble CD14, S100 calcium binding protein A13, and 14-3-3 protein theta (YWHAQ). Levels of COMP and HA were also measured in the plasma of these patients. To find predictors of postoperative function, multivariable regression analyses were performed.

**Results:**

Proteomic analyses highlighted YWHAQ and LYVE-1 as being differentially abundant between the clinical responders/improvers and nonresponders after microfracture. A linear regression model after backward variable selection could relate preoperative concentrations of SF proteins (HA, YWHAQ, LYVE-1), activity of ADAMTS-4, and patient demographic characteristics (smoker status and sex) with Lysholm score 12 months after microfracture. Further, a generalized linear model with elastic net penalization indicated that lower preoperative activity of ADAMTS-4 in SF, being a nonsmoker, and being younger at the time of operation were indicative of a higher postoperative Lysholm score (improved joint function) after osteotomy surgery.

**Conclusion:**

We have identified biomarkers and generated regression models with the potential to predict clinical outcome in patients treated with microfracture or osteotomy of the knee.

**Clinical Relevance:**

Candidate protein biomarkers identified in this study have the potential to help determine which patients will be best suited to treatment with microfracture or osteotomy.

Some of the most widely used surgical approaches that aim to repair chondral or osteochondral defects of the knee include microfracture and osteotomy.^10,41^ Although advanced cell therapy approaches such as autologous chondrocyte implantation (ACI) have been highlighted as more efficacious and cost-effective for repair of chondral and osteochondral defects of the knee,^26^ these surgeries are not available in nonspecialist orthopaedic centers and require access to Good Manufacturing Practice (GMP) facilities able to culture expand patients’ cells; hence, alternative surgeries such as microfracture and osteotomy are often used. Microfracture and osteotomy can be economical treatments, but only for those individuals in whom these surgeries are successful; therefore, there is a need for early identification of patients who are likely to benefit from microfracture and osteotomy. The Osteoarthritis Research Society International (OARSI) previously highlighted the need to identify biomarkers that can predict which patients are most likely to benefit from surgical interventions to delay or prevent the development of osteoarthritis (OA),^16^ and we are unaware of any published studies that have addressed this need for microfracture or osteotomy.

Work to identify biomarkers (biochemical and imaging) for the diagnosis and prognosis of OA has been abundant and ongoing for many years.^13^ However, far fewer studies have focused on the identification of biomarkers to predict clinical outcome in response to a surgical intervention to prevent or delay the onset of OA. To our knowledge, the only published studies that have used human samples with this aim have been related to ACI^11,12,45^ or anterior cruciate ligament (ACL) reconstruction.^18^ Wright et al^45^ demonstrated that assessment of proteins of known biological relevance to OA within the synovial fluid (SF) can identify protein markers that, when combined with known demographic or surgical risk factors, can be used to predict the success of surgical treatment with ACI. In support for this approach, Latterman et al^18^ demonstrated that assessment of OA-related proteins, as well as inflammatory cytokines, in SF collected at the time of surgery could be used to differentiate between individuals who clinically improved and those who did not at 2 years after ACL reconstruction. As alternatives to assessing OA-related proteins, unbiased discovery-based approaches have been used to identify within-treatment proteome shifts that are unique to ACI nonresponders.^11,12^ In the study described here, we used both these targeted and nontargeted approaches. We hypothesized that these approaches can be used to identify protein biomarkers that may predict an individual’s probability of a successful outcome in response to microfracture or osteotomy.

Proteins were selected for our targeted analysis that have known biological relevance to cartilage degeneration/turnover and OA, along with proteins that have previously been highlighted as candidate biomarkers for the prediction of outcome to ACI via nontargeted proteomic analyses.^11,12^ The cartilage matrix–related proteins and enzymes assessed in this study were cartilage oligomeric matrix protein (COMP), hyaluronan (HA), A disintegrin and metalloproteinase with thrombospondin motifs 4 (ADAMTS-4; aggrecanase-1), and matrix metalloproteinases 1 and 3 (MMP-1 and MMP-3). COMP is a noncollagenous glycoprotein involved in collagen-collagen interactions.^43^ HA is a glycosaminoglycan that constitutes a key component of the cartilage extracellular matrix.^37^ Both COMP and HA have long been suggested as prognostic biomarkers of OA progression.^37,38^ ADAMTS-4, MMP-1, and MMP-3 are key enzymes in regulating cartilage homeostasis. ADAMTS-4 cleaves large chondroitin sulphate HA-binding proteoglycans including aggrecan.^34^ MMPs also break down other key cartilage matrix components, specifically collagen II by MMP-1 and a broad range of matrix components including aggrecan by MMP-3.^24,25^ Soluble CD14 (sCD14) was assessed, as this protein has been associated with OA progression and pain.^8^ CD14 is found on the surface of monocytes and macrophages and regulates the production of a number of inflammatory mediators.^8^ S100 calcium binding protein A13 (S100A13) was also assessed, as this protein has previously been highlighted as having potential to predict ACI outcome after untargeted proteomic analysis.^12^ S100A13 is a member of the S100 family of proteins, which have a wide variety of extracellular functions, many of which act as alarmins that contribute to the regulation of immune and inflammatory responses and posttraumatic injury respones^46^; however, no specific relation between S100A13 and the joint environment homeostasis or inflammatory response has been reported.

Within the field of OA, much of the work aimed at identifying prognostic and predictive biomarkers has relied on SF because this fluid represents the whole joint environment. However, identification of protein biomarkers that can be assessed within the blood rather than the SF remains the ultimate goal in clinical practice, as this would provide a less invasive method of predicting which individuals are likely to benefit from surgical procedures to treat cartilage defects. Therefore, we have also assessed some commonly studied OA-related biomarker proteins within the plasma of patients who have undergone osteotomy and microfracture.

## Methods

### Patients

After local research ethics committee approval was granted (11/NW/0875 and 06/Q6201/9; see declarations) and with informed consent of patients, blood and SF samples were collected from individuals undergoing osteotomy or microfracture at our center between 2015 and 2019, along with samples collected in our research group’s biobank since 2006. Before surgery, patients’ demographic details were recorded, and their functional status was determined via completion of the modified Lysholm patient-reported outcome measure.^22,39^ The modified Lysholm score ranges from 0 to 100, with 100 representing “perfect” knee function.^22^ Individuals were deemed to respond to surgery if they demonstrated a minimal clinically important difference (MCID) of a 10-point increase in Lysholm score between pre- and postoperative assessments.^9,33,36^ Postoperative scores were collected at approximately 12 months after surgery (median ± interquartile range, 13 ± 6.5 months). The severity of a patient’s OA before surgery was determined using Kellgren-Lawrence grading of radiographs^14^ independently by 2 consultant orthopaedic surgeons (P.G., P.J.). A mean of the 2 surgeons’ scores was taken as the preoperative Kellgren-Lawrence score. The details of these patients are described in Table 1. Samples from the whole patient cohort were assessed for all biomarker proteins including those related to OA and cartilage biology, and to validate those proteins identified in this study via exploratory proteomic analysis (Table 1).

**Table 1:** Demographic Characteristics and Biomarker Data for Participants Treated With Either Osteotomy or Microfracture^*a*^

	Microfracture (n = 19)	Osteotomy (n = 13)
Patient characteristics		
Age, y	34 (22) [17-67]	46 (9) [31-58]
Male, n	12	8
Body mass index	25 (5) [19-36]	30 (2) [21-34]
Baseline Lysholm score	50 (30) [23-88]	52 (17) [17-66]
Postoperative Lysholm score	54 (35) [16-96]	79 (33) [38-92]
Baseline Kellgren-Lawrence score	0.5 (1) [0-2]	2 (1) [1-3]
Treatment side: right leg, n	9	6
Treatment side: left leg, n	10	7
Smokers, n	1	2
Synovial fluid markers		
COMP, mg·mL^-1^	157.3 (90.3) [29.1-383.2]	147.1 (129.8) [44.5-310.6]
HA, mg·mL^-1^	17.7 (9.1) [3.1-34.0]	14.2 (23.1) [1.6-48.9]
sCD14, ng·mL^-1^	765.6 (1516.9) [11.3-5014.7]	2308.4 (2144.2) [479.1-3880.2]
ADAMTS-4/aggrecanase-1, ng·mL^-1^	0.4 (21.4) [0.4-44.0]	0.4 (17.4) [0.4-34]
MMP-1, ng·mL^-1^	0.3 (5.3) [0.04-22.9]	1.7 (7.9) [0.3-10.1]
MMP-3, ng·mL^-1^	136.2 (205.2) [0-5060.9]	532.7 (515.8) [163.8-2867.8]
S100A13, pg·mL^-1^	2529.4 (2116.3) [946.5-6795.6]	1552.5 (2723.2) [0-5807.8]
YWHAQ, ng·mL^-1^	0.5 (0.5) [0.5-10.6]	1.5 (2.0) [0.5-7.98]
LYVE-1, ng·mL^-1^	4.1 (4.1) [1.5-16.1]	1.1 (1.5) [0.5-5.3]
Plasma markers		
COMP, ng·mL^-1^	491.4 (155.3) [342.4-758.2]	629.5 (230.7) [342.4-1212.0]
HA, ng·mL^-1^	0.5 (5.0) [0.5-20.9]	0.5(16.2) [0.5-57.8]

a Values are expressed as median (interquartile range) [range] unless otherwise noted. ADAMTS-4, A disintegrin and metalloproteinase with thrombospondin motifs 4; COMP, cartilage oligomeric matrix protein; HA, hyaluronan; LYVE-1, lymphatic vessel endothelial hyaluronan receptor 1; sCD14, soluble CD14; S100A13, S100 calcium binding protein A13; YWHAQ, 14-3-3 protein theta.

A group of samples were selected for proteomic analysis from a subgroup of patients who demonstrated the greatest improvement in clinical score (responders) or the least improvement or a worsening of function (nonresponders) at 12 ± 2 months after osteotomy or microfracture. Other selection criteria for the proteomic study included having >2 mL of SF sample for analysis; no blood contamination staining of the SF, as this has been demonstrated to alter the detection of proteins within the fluid^44^; and a dilution factor of <14 (detailed in Table 2).

**Table 2:** Demographic Data for Participants Whose Synovial Fluid Samples Were Used for Proteomic Analysis and Who Responded Clinically (Responders) or Who Did Not Respond (Nonresponders) to Either Osteotomy or Microfracture^*a*^

	Osteotomy			Microfracture		
	Responders (n = 3)	Nonresponders (n = 4)	*P* Value R vs NR	Responders (n = 3)	Nonresponders (n = 3)	*P* Value R vs NR
Difference in Lysholm score	44 (21 to 62)	−8 (0 to −16)	.004	36 (16 to 72)	−3 (0 to −4)	.070
Baseline Kellgren-Lawrence score	2 (1.5 to 2.5)	2.5 (1 to 3)	.465	00 (0 to 0)	1 (1 to 2)	.070
Age, y	44 (29 to 59)	40 (31 to 46)	.807	25 (19 to 34)	38 (24 to 46)	.201
Male, n	3	2	>.999	1	2	>.999
Body mass index	34 (30 to 35)	30 (28 to 30)	.053	20 (19 to 21)	34 (29 to 38)	.095
Treatment side: right, n	1	2	>.999	2	3	>.999
Treatment side: left, n	2	2	>.999	1	0	>.999
Smoker, n	0	1	>.999	1	0	.400
Dilution factor of synovial fluid	4 (0 to 9)	7 (3 to 13)	.856	7 (6 to 8)	7 (6 to 10)	.877

a Data are shown as median (range) unless otherwise indicated. None of the demographic parameters, other than difference in Lysholm scores, showed differences between responders (R) and nonresponders (NR) in individuals whose synovial fluids were compared for each surgical procedure (Lysholm, age, body mass index, dilution factor: unpaired *t* test; sex, treatment side, smoker status: Mann-Whitney *U* test).

### SF and Plasma Collection and Storage

SF was collected from patients’ knee joints immediately before microfracture or osteotomy surgery by injecting 20 mL of saline and then extending and flexing the leg at least 20 times before intra-articular aspiration of as much SF as possible.^32^ At this time, blood samples were also collected by venipuncture. SF and plasma were then centrifuged at 6000*g* for 15 minutes at 4°C, and the supernatant was removed before being divided into aliquots and stored in −196°C liquid nitrogen before analyses.

### Proteomic Analysis of SFs

#### Sample Preparation and Analysis Using Proteomics

SF samples were not pooled at any point of the proteomic sample preparation or mass spectrometry stages; therefore, the protein abundance was quantified for each of the 13 samples, and mean protein abundance for each experimental group was calculated before the analysis of protein changes.

#### SF Preparation and Protein Equalization Using ProteoMiner

ProteoMiner beads (BioRad) were used to compress the dynamic range of proteins to allow improved identification of low-abundance proteins, as described previously.^12,28^ The total protein concentration of hyaluronidase (1 mg·mL^-1^)− treated SFs^12^ was quantitated using a Pierce 660-nm protein assay (Thermo Scientific). Next, 5 mg of total protein was incubated with ProteoMiner beads following kit instructions. Proteins attached to the beads were then treated with 0.05% (wt/vol) RapiGest (Waters) in 25 mM ammonium bicarbonate for 10 minutes at 80°C before reduction, alkylation, and in situ protein digestion, which was carried out using trypsin in LoBind protein tubes (Eppendorf) without removal of the beads. Samples were acidified using trifluoroacetic acid to a final concentration of 0.5% (vol/vol) and multiple centrifugation steps to inactivate and precipitate the RapiGest detergent. Peptide-containing supernatant fractions were then frozen at −20°C before being analyzed using liquid chromatography−tandem mass spectrometry (LC-MS/MS).

#### LC-MS/MS and Label-Free Quantification

A NanoAcquity ultraperformance LC (Waters) coupled online to a Q-Exactive Quadrupole-Orbitrap instrument (Thermo-Fisher Scientific) was used to analyze tryptic peptides on a 2-hour gradient.^12,28^ For label-free quantification, raw files of the acquired data were analyzed (as described previously^12^) in ProgenesisQI software (Waters),^27^ and the top 5 spectra for each feature were used for peptide identification in a locally implemented Mascot server (Version 2.3.01), searching against the Unihuman Reviewed database. Peptide matches above an identified threshold were adjusted to give a false discovery rate of 1% before the protein identifications were reimported into ProgenesisQI for the label-free relative quantification of unique peptides. Statistical analysis was performed using ProgenesisQI software; in brief, transformed normalized abundances were used for 1-way analysis of variance, and all peptides (with *P* < .05) of an identified protein were included. To select proteins for biochemical validation, the mean abundance of each protein in each of the experimental groups was calculated, and significant proteins (false discovery rate; *P* < .05) with a ≥±2.0-fold change between comparator groups were reported.

### Assessment of Protein Abundances Using Enzyme-Linked Immunosorbent Assay (ELISA)

#### Assessment of Proteins Identified in the LC-MS/MS Proteomic Analyses Using ELISA

We selected 14-3-3 protein theta (YWHAQ) to validate the LC-MS/MS findings, as this protein was detected only in nonresponders and was not detected at all in responders to microfracture, deeming it an ideal candidate biomarker. Lymphatic vessel endothelial hyaluronan receptor 1 (LYVE-1) was also selected to validate the LC-MS/MS findings because it can bind to HA,^47^ a key component of articular cartilage and SF. LYVE-1 is also highly expressed in the synovium of OA patients who have synovial villous hypertrophy and chronic inflammatory cell infiltrate.^47^ YWHAQ and LYVE-1 were quantified using ELISAs (YWHAQ, Cusabio; LYVE-1, R&D Systems) according to the manufacturers’ instructions. SF samples from the same patients as were assessed in the LC-MS/MS analysis were used to validate the proteomic findings. These samples were assessed in duplicate, and mean optical density values were used to calculate the protein concentration. SF was diluted 1:50 for the assessment of LYVE-1 and was assayed neat (ie, not further diluted above the dilution due to lavage) for YWHAQ. The concentration of each protein was normalized to total protein concentration, to validate the LC-MS/MS, as equal concentrations of protein were loaded onto the ProteoMiner beads. Statistical analysis was performed in GraphPad Prism Version 8.0. These proteins were then included in “targeted” biomarker analyses in the larger cohort of patients (Table 1) and were included in multivariable regression modeling to determine whether they had any predictive value over and above other proteins.

#### Assessment of OA- and Cartilage-Associated Proteins Using ELISA

Targeted analyses of proteins that are associated with the development of OA and with known cartilage biology were performed using ELISA. These included proteins that we have previously highlighted as associated with clinical outcome in patients undergoing cell therapy via ACI for the treatment of early OA.^11,12,45^ Levels of COMP and HA in both the SF and the plasma were assessed using an ELISA (BioVendor Laboratory Medicine) and an enzyme-linked protein binding assay (Corgenix), respectively, as described previously.^45^ As in our previous work, SF concentrations of sCD14 were measured using a Quantikine ELISA kit (Biotechne), and ADAMTS-4 (aggrecanase-1) enzyme activity assessed using an endpoint fluorometric substrate assay (SensoLyte 520 aggrecanase-1 Assay Kit; AnaSpec).^45^ Concentrations of MMP-1, MMP-3, and S100A13 were assayed using duo-sets (MMP-1 and S100A13; Biotechne) or Quantikine ELISAs (MMP-3; Biotechne).^11,12^ All assays were optimized to determine the appropriate sample dilution factor (over and above dilution due to lavage) as follows: ADAMTS-4, undiluted; sCD14, 1:200; COMP, 1:1000; HA, 1:3000; MMP-1, 1:3; MMP-3, 1:100; and S100A13, 1:20. Plasma samples were diluted 1:50 for COMP and assayed neat for HA. The concentration of each protein was normalized to the dilution factor of the SF. To determine the dilution of the SF (due to lavage), urea concentrations in the SF and plasma were assessed using a QuantiChrom urea assay kit (Universal Biologicals), and SF biomarker values were normalized to the urea concentration in blood plasma as described previously.^17^ The principle of this method to determine dilution factor is based on the strong correlation between the concentration of urea in the SF and plasma of an individual; therefore, the SF volume can be calculated by assessing matched patient SF and plasma samples.^17^ Median baseline protein biomarker levels are summarized in Table 1.

### Statistical Analysis

Data analyses were performed using the statistical programming language R Version 4.0.2.^31^ Independent models were built for both the total microfracture (n = 19) and the osteotomy (n = 13) cohorts. The postoperative Lysholm score was used as the dependent variable, and independent variables were preoperative Lysholm score, patient age at the time of microfracture or osteotomy, body mass index, smoker status, and baseline level of the 9 SF biomarkers (COMP, HA, sCD14, ADAMTS-4, MMP-1, MMP-3, S100A13, LYVE-1, and YWHAQ) and the 2 plasma biomarkers (HA and COMP). Imputed values were used when there were nondetections for any of the proteins assessed using ELISA.^45^ For sCD14, plasma HA, and YWHAQ, the imputed value was taken as equal to (1/√2) times the lowest detected value (sCD14, 11.3; plasma HA, 0.5; YWHAQ, 0.5), which was the value applied to ADAMTS-4 nondetections in our previous study (0.40).^45^ Any missing data were imputed using multiple imputation by chained equations.^1^ The number of model features compared with the number of observations was relatively high, and therefore linear regression with elastic net penalization was performed. Elastic net penalization is a combination of least absolute shrinkage and selection operator (LASSO) and ridge regression. LASSO penalization shrinks each predictor differently and allows variables to be removed entirely by shrinking coefficients to zero,^42,50^ whereas for ridge regression, the penalty term shrinks the effect of the predictor equally and none are reduced to zero. Thereby, elastic net can eliminate features entirely and allows for reduction of the effect of less important model features.

To provide confidence in the findings and to build models that best represented the different datasets, multivariable linear regression models were built and variables selected using backward variable selection. Backward elimination removes the least significant effect that does not meet the level for remaining in the model, and this is repeated until no other effect in the model meets the specified level for removal. This method allows one to account for collinearity between variables.^6^

Protein markers that demonstrated a significant contribution to the models (*P* < .05) were then assessed for their potential to predict outcome to surgery based on the “responder” definitions given previously, specifically an MCID in Lysholm score of at least 10 points at 12 months after surgery. When patients were categorized as responders and nonresponders, a small number of patients remained in each response arm for the 2 different surgeries. Therefore, to assess whether there were differences in the activity or expression of these enzymes and proteins, Mann-Whitney tests were performed using GraphPad Prism Version 8.0.

## Results

### Patients

From the overall cohort, 13 patients were selected for proteomic analysis of the SF, based on the selection criteria detailed in the Methods section. After osteotomy, 3 donors were considered extreme responders with a mean improvement of 44 Lysholm points (range, 21-62 points), and 4 donors were considered nonresponders with a mean decrease in Lysholm score of 8 points (range, 0-16 points). After microfracture, 3 donors were deemed extreme responders (mean improvement of 26 Lysholm points; range, 16-72 points), and a further 3 donors were deemed extreme nonresponders (mean decrease of 3 Lysholm points; range 0-4, points). The demographic information for these patients is shown in Table 2.

To assess candidate biomarkers in a larger cohort, this study included 13 patients undergoing osteotomy and 19 patients undergoing microfracture (Table 1). In osteotomy patients, the mean ± SD baseline Lysholm score was 49.0 ± 13.9 points, which improved to 72.0 ± 21.0 points at 16 ± 8.9 months after osteotomy surgery. In microfracture patients, the mean ± SD baseline Lysholm score was 50.7 ± 18.7 points, which improved to only 58.7 ± 25.6 points at 15.2 ± 9.6 months postoperatively. When patients were defined as responders or nonresponders to surgery based on an MCID of 10 Lysholm points, 8 individuals who underwent osteotomy were responders, with a median increase in Lysholm points of 37 points (range, 16 to 62 points), and 5 individuals who had osteotomy were nonresponders, with a median Lysholm point change of −4 points (range, 0 to −16 points). Further, 8 patients who had microfracture surgery were responders, with a median Lysholm point increase of 23 points (range, 12 to 72 points), and 11 who had microfracture surgery were nonresponders, with a median Lysholm point change of −3 points (range, 8 to −34 points). This study entailed samples from 20 male patients (8 osteotomy and 12 microfracture) and 12 female patients, aged between 17 and 67 years at the time of surgery. Table 1 details the demographic variables of these patients.

### Differential Abundance of SF Proteins in Nonresponders Compared With Responders to Microfracture Identified Using Proteomic Analysis

Individuals who did not respond well clinically to microfracture demonstrated a differential baseline proteome compared with those who responded well. We found that 30 proteins were differentially abundant (±2.0-fold; *P* < .05) in the preoperative SF of responders compared with nonresponders of microfracture (Table 3). YWHAQ was present in the SF of only the selected individuals who did not respond to microfracture, and no presence of this protein was detected using LC-MS/MS in the responders. Conversely, small ubiquitin-related modifier 4 (SUMO4) was detected only in the SF of responders to microfracture and not in the nonresponders. LYVE-1, a protein that was of biological interest because it can bind to HA,^47^ was 6-fold higher in the selected microfracture responders compared with nonresponders.

**Table 3:** Fold Change of Proteins That Were Differentially Abundant (±2.0-fold; false discovery rate, *P* < .05) in the Preoperative Synovial Fluid of Individuals Who Did Not Improve After Microfracture (Nonresponders; n = 3) Compared With Those Who Did Improve (Responders; n = 3)^*a*^

Protein		
Description	Accession	Fold Change
Small ubiquitin-related modifier 4 (SUMO4)	Q6EEV6	Infinity
55-kDa erythrocyte membrane protein	Q00013	−2103005.6
Protein argonaute 1	Q9UL18	−147.4
Complement C1r subcomponent-like protein	Q9NZP8	−11.0
Lymphatic vessel endothelial hyaluronic acid receptor 1 (LYVE-1)	Q9Y5Y7	−6.0
Peroxiredoxin 4	Q13162	−4.4
Adiponectin	Q15848	−2.8
IgGFc-binding protein	Q9Y6R7	−2.7
Ubiquitin-conjugating enzyme E2 variant 1	Q13404	−2.5
Alpha-2–macroglobulin	P01023	−2.4
Lysosomal acid phosphatase	P11117	−2.1
Alpha-lactalbumin	P00709	2.0
Coagulation factor XI	P03951	2.1
Beta-Ala-His dipeptidase	Q96KN2	2.2
Ficolin 3	O75636	2.5
Retinol-binding protein 4	P02753	2.5
Serine protease HTRA1	Q92743	2.6
28S ribosomal protein S34, mitochondrial	P82930	2.9
Peptidyl-glycine alpha-amidating monooxygenase	P19021	3.1
Dickkopf-related protein 3	Q9UBP4	3.1
Serum amyloid P-component	P02743	3.1
Ribonuclease 4	P34096	3.3
Insulin-like growth factor II	P01344	3.6
eIF-2-alpha kinase activator GCN1	Q92616	5.2
Glycosylphosphatidylinositol anchor attachment 1 protein	O43292	5.4
Mannan-binding lectin serine protease 2	O00187	6.8
Poly(rC)-binding protein 3	P57721	10.7
Sodium channel protein type 8 subunit alpha	Q9UQD0	11.8
Platelet factor 4	P02776	44.4
14-3-3 protein theta (YWHAQ)	P27348	Infinity

a Positive numbers denote an increase in the protein in nonresponders; negative numbers denote an increase in the protein in responders.

### Differential Abundance of SF Proteins in Nonresponders Compared With Responders to Osteotomy Identified Using Proteomic Analysis

An SF proteome shift was found between individuals who did or did not respond well to osteotomy. Table 4 demonstrates the 15 proteins that were differentially abundant (±2.0-fold; *P* < .05) in the SF between the 2 different clinical outcome groups. Both YWHAQ and an undetectable protein KIAA1107 demonstrated a much higher abundance in the nonresponders to osteotomy compared with the responders, with YWHAQ again being undetected in the responders via LC-MS/MS.

**Table 4:** Fold Change of Proteins That Were Differentially Abundant (±2.0-fold; false discovery rate, *P* < .05) in the Preoperative Synovial Fluid of Individuals Who Did Not Improve After Osteotomy (Nonresponders; n = 4) Compared With Those Who Did Improve (Responders; n = 3)^*a*^

Protein		
Description	Accession	Fold Change
Integrin alpha-M	P11215	−5.0
Cubilin	O60494	−4.6
AT-rich interactive domain-containing protein 5B	Q14865	−4.3
Filamin-A	P21333	−4.1
Cystatin-C	P01034	−3.9
Immunoglobulin kappa variable 3D-20	A0A0C4DH25	−2.9
Ubiquitin-conjugating enzyme E2 variant 1	Q13404	−2.8
Immunoglobulin heavy constant alpha 1	P01876	−2.7
Serum amyloid A-2 protein	P0DJI9	−2.3
Immunoglobulin kappa variable 1-6	A0A0C4DH72	−2.1
Hyaluronan and proteoglycan link protein 1	P10915	2.2
Insulin-like growth factor-binding protein 5	P24593	7.3
von Willebrand factor	P04275	9.7
Uncharacterized protein KIAA1107	Q9UPP5	263.0
14-3-3 protein theta (YWHAQ)	P27348	Infinity

a Positive numbers denote an increase in the protein in nonresponders; negative numbers denote an increase in the protein in responders.

### Biochemical Validation of Proteomic Analyses

YWHAQ was confirmed to have undetectable concentrations in microfracture responders and detectable concentrations only in microfracture nonresponders when measured using ELISA (Appendix Figure A1, available in the online version of this article). We also assessed YWHAQ using ELISA in the osteotomy samples used for the proteomic analyses but found that for 2 of the responder samples, detectable levels were measured using ELISA, whereas they were not detected at all via LC-MS/MS (data not shown).

Further, the proteomic finding that LYVE-1 demonstrated greater abundance in microfracture responders compared with nonresponders could be confirmed using biochemical analyses (mean ± SD: responders, 154.6 ± 45.0 pg·mL^-1^/mg protein; nonresponders, 40.4 ± 14.6 pg·mL^-1^/mg protein; *P* = .01) (Appendix Figure A1, available online). These proteins were therefore included in further studies to assess their predictive potential compared with other biomarkers and demographic data in a larger patient cohort.

### Biomarker Levels

SF and plasma samples were included from all 32 patients for analysis of targeted OA-related biomarkers and candidate markers identified in our nontargeted proteomic study. The mean ± SEM SF dilution factor was 7.8 ± 7.7, with the mean dilution factor being 5.3 ± 4.6 and 5.6 ± 4.5 in osteotomy responders and nonresponders and 9.3 ± 6.9 and 8.4 ± 10.2 in microfracture responders and nonresponders, respectively. Three of the SF proteins (sCD14, ADAMTS-4, and YWHAQ) and HA in plasma had samples with undetectable levels, and values were imputed as described in the Methods section.

### Multivariable Linear Models of Predictors of Postoperative Lysholm Score After Surgical Treatment

#### Multivariable Linear Models for Microfracture Outcome

Generalized linear models (GLMs) with elastic net penalization were built using the postoperative Lysholm score after microfracture (Appendix Table A1, available online). For the GLM of microfracture postoperative Lysholm score, the *r*^2^ was low (0.5) and the root mean square error (RMSE) was high (26.4). Therefore, a linear regression model was generated after backward variable selection; this model better related the selected variables with the dependent variable, postoperative Lysholm score (*r*^2^ = 0.71; RMSE = 13.6) (Table 5). A number of model parameters significantly contributed to the linear regression model for microfracture outcomes, which were baseline levels of SF HA, YWHAQ, plasma HA, ADAMTS-4, LYVE-1, and patient smoker status and sex (Table 5). Although the *r*^2^ was low for the GLM with elastic net penalization for the postmicrofracture Lysholm score (Appendix Table A1), HA was the variable that most strongly contributed to the model’s prediction value, again highlighting this cartilage matrix protein as a predictor of clinical outcome after microfracture.

**Table 5:** Linear Regression Model After Backward Variable Selection for Predictors of the Postmicrofracture Lysholm Score (n = 19)^*a*^

Component	Regression Coefficient (SEM)	Adjusted *r*^2^ Value	RMSE	*P* Value
Total model		0.71	13.6	.04
SF HA	−3.0 (0.6)			.003
YWHAQ	−8.09 (1.7)			.003
Plasma HA	−3.9 (1.1)			.01
Smoker	−49.9 (14.6)			.01
Sex	−27.3 (8.0)			.02
ADAMTS-4	1.0 (0.35)			.03
LYVE-1	0.001 (0.0)			.04
Baseline Lysholm score	−0.81 (0.3)			.05
Kellgren-Lawrence score	17.5 (7.3)			.05
sCD14	0.01 (0.0)			.09
MMP-3	0.01 (0.0)			.17
Body mass index	−0.66 (0.7)			.38
MMP-1	−0.71 (0.8)			.41

a ADAMTS-4, A disintegrin and metalloproteinase with thrombospondin motifs 4 (ng·mL^-1^); LYVE-1, lymphatic vessel endothelial hyaluronan receptor 1 (ng·mL^-1^); MMP-1, matrix metalloproteinase 1 (ng·mL^-1^); MMP-3, matrix metalloproteinase 3 (ng·mL^-1^); plasma HA, plasma hyaluronan (ng·mL^-1^); RMSE, root mean square error; sCD14, soluble CD14 (ng·mL^-1^); SF HA, synovial fluid hyaluronan (mg·mL^-1^); YWHAQ, 14-3-3 protein theta (ng·mL^-1^).

#### Multivariable Linear Models for Osteotomy Outcome

A GLM with elastic net penalization was built using the postoperative Lysholm after osteotomy (Table 6), which had a strong correlation coefficient (*r*^2^ = 0.77) and a low RMSE (12.1). The GLM with elastic net penalization of postoperative Lysholm after osteotomy determined that the variables that most strongly contributed to the model were smoker status, age at time of operation, and preoperative ADAMTS-4 activity (Table 6). These findings indicate that being a nonsmoker and being younger at the time of operation were related to an increased Lysholm score after osteotomy surgery.

**Table 6:** Generalized Linear Regression Model With Elastic Net Penalization for Predictors of the Postosteotomy Lysholm Score (n = 13)^*a*^

Component	*r*^2^ Value	RMSE	Variable Importance
Total model	0.77	12.1	
Smoker			100
Age			3
ADAMTS-4			2

a Final elastic net model parameters were alpha 0.6, lambda 5.2. Alpha is a value between 0 and 1, where 0 is pure ridge regression, 1 is pure LASSO (least absolute shrinkage and selection operator), and values between are a mixture of both. Lambda is the shrinkage factor applied to model coefficients. ADAMTS-4, A disintegrin and metalloproteinase with thrombospondin motifs 4 (ng·mL^-1^); RMSE, root mean square error.

#### Assessment of Candidate Predictors in Relation to a 10-Point Improvement in the Lysholm Score After Surgical Treatment

The biomarker proteins that significantly contributed to the predictive models of microfracture or osteotomy outcome were assessed to determine whether there was a differential concentration between individuals who were deemed responders or nonresponders to either surgery.

**Figure 1.**
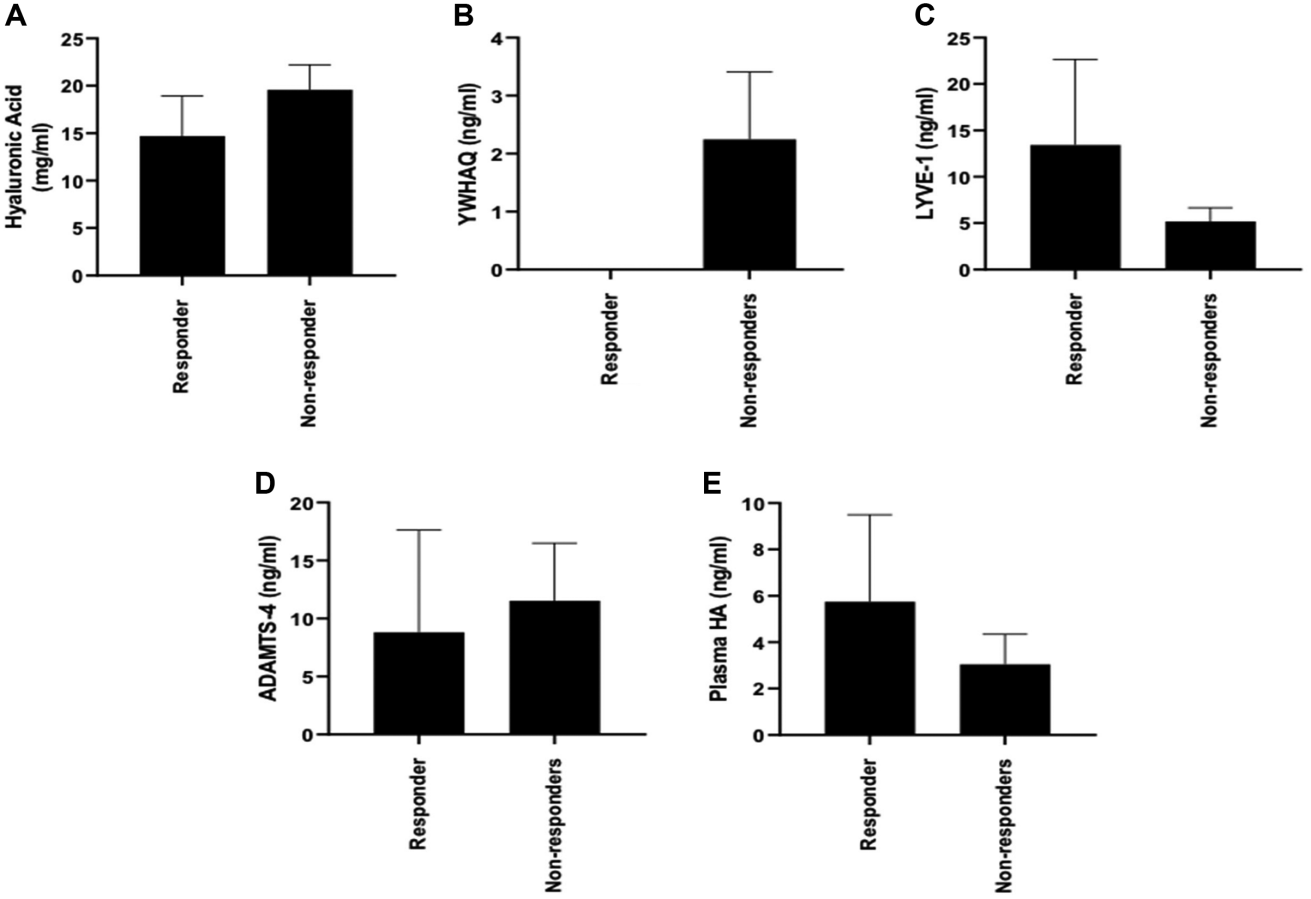
Assessment of differential abundance/activity of candidate predictive markers in responders (n = 8) compared with nonresponders (n = 11) to microfracture surgery. Concentrations (mean ± SEM) of (A) hyaluronic acid (responders, 14.7 ± 4.2 mg·mL^-1^; nonresponders, 19.6 ± 2.6 mg·mL^-1^; *P* = .52), (B) 14-3-3 protein theta (YWHAQ) (responders, 0 ± 0 ng·mL^-1^; nonresponders, 2.5 ± 1.3 ng·mL^-1^; *P* = .18), and (C) lymphatic vessel endothelial hyaluronan receptor 1 (LYVE-1) (responders, 13.4 ± 9.2 ng·mL^-1^; nonresponders, 5.2 ± 1.5 ng·mL^-1^; *P* = .918) in the synovial fluid were not different (Mann-Whitney) between responders and nonresponders to microfracture surgery. (D) Enzyme activity of A disintegrin and metalloproteinase with thrombospondin motifs 4 (ADAMTS-4) was not significantly different between responders (8.8 ± 8.8 ng·mL^-1^) and nonresponders (11.5 ± 5.0 ng·mL^-1^) to microfracture surgery (Mann-Whitney; *P* = .76). (E) Plasma hyaluronan (HA) concentration was not different between microfracture responders (5.7 ± 3.8 ng·mL^-1^) and nonresponders (3.0 ± 1.3 ng·mL^-1^) (Mann-Whitney; *P* > .99).

We found that 8 of the patients responded to microfracture surgery and 11 were nonresponders based on an MCID of 10 Lysholm points. Figure 1 demonstrates that the SF concentration of none of the proteins (HA, YWHAQ, ADAMTS-4, LYVE-1) that contributed to the predictive model of microfracture outcome was significantly different between microfracture responders and nonresponders. The concentration of YWHAQ was not significantly different (*P* = .18; Mann-Whitney) between responders (mean ± SEM, 0 ± 0 ng·mL^-1^) and nonresponders (2.5 ± 1.3 ng·mL^-1^) to microfracture. However, in all of the responders to microfracture surgery, YWHAQ was undetectable (n = 8) when assessed using ELISA, but in clinical nonresponders (n = 11), YWHAQ was detected in only 50% of cases. Thus, assessment of YWHAQ in the total patient cohort confirms the findings of the proteomic analysis that YWHAQ is undetectable in responders to microfracture surgery. The concentration of HA within the plasma was not significantly different between the microfracture responders and nonresponders (*P* > .99).

**Figure 2.**
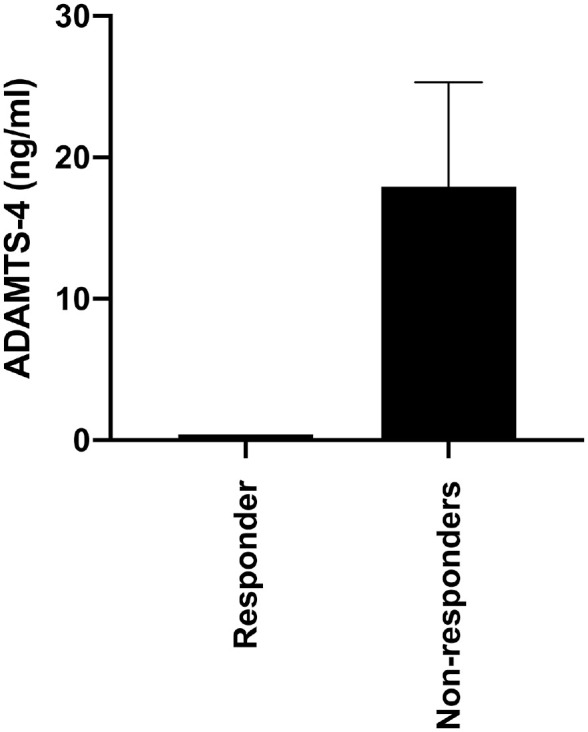
None of the responders to osteotomy surgery demonstrated detectable enzyme activity of A disintegrin and metalloproteinase with thrombospondin motifs 4 (ADAMTS-4); however, enzyme activity was not significantly different between the responders (n = 8) compared with nonresponders (n = 5) (responders, 0.4 ± 0 ng·mL^-1^; nonresponders, 17.9 ± 7.3 ng·mL^-1^; Mann-Whitney; *P* = .17). Data are expressed as mean ± SEM.

We found that 8 of the 13 patients treated with osteotomy improved by at least 10 points in the Lysholm score after 1 year and were therefore classed as responders. The activity of ADAMTS-4 was not significantly different between individuals who responded and did not respond to osteotomy (Figure 2) (mean ± SEM: responders, 0.4 ± 0 ng·mL^-1^; nonresponders, 17.9 ± 7.3 ng·mL^-1^; Mann-Whitney; *P* < .05). However, all of the individuals who responded to osteotomy had undetectable activity of this enzyme within their SFs, whereas activity was detectable in 3 of the 5 nonresponders.

## Discussion

Identification of predictive biomarkers that are able to recognize individuals who are most likely to receive clinical benefit from interventions aimed at delaying or preventing the development of OA has been highlighted as a research priority by the OARSI.^16^ The current study aimed to identify novel candidate biomarkers with the potential to predict outcomes of 2 of the most commonly used clinical procedures, microfracture and osteotomy. This is the first study, to our knowledge, to assess a panel of protein markers for their potential to predict patient outcomes of these surgeries. We have previously identified candidate protein markers for the prediction of clinical outcomes of the cell therapy ACI.^45^ However, the work described here has the potential for more widespread application because microfracture and osteotomy surgeries are offered at a large number of hospitals, with >100,000 microfracture procedures in knees, hips, or shoulders estimated to be carried out per year globally.^7^ These procedures provide less expensive, alternative surgical options for the treatment of joint damage and early OA, which, if used to treat the patients who are likely to benefit, can reduce the number of patients who will develop end-stage OA.

To identify novel candidate biomarkers, we used a 2-pronged approach. The first was to use label-free quantitation proteomics in a nontargeted approach with the aim of identifying completely novel biomarkers that had not previously been associated with OA or cartilage damage/repair. The second, targeted approach was to assess a panel of SF and plasma proteins that we routinely test and that have been previously associated with OA^32,45^ or that we have identified as changed in response to ACI.^11,12^ This method meant that any candidate proteins identified from the proteomic analysis could be assessed in a larger cohort to determine whether they added any predictive value over and above other more commonly assessed OA proteins, baseline OA severity (as deemed by Kellgren-Lawrence score), and patient demographic characteristics, together contributing to the development of predictive models for patient outcome to microfracture or osteotomy surgery. Further, particularly for the microfracture cohort, there was a wide range of patient ages, which in itself could account for differential abundance of some of the measured proteins (eg, serum HA^19^); therefore, this statistical approach could account for collinearity between parameters, such as between age and serum HA.

One of the limitations of our study is that only a small number of samples were used in the proteomic analysis. This is because we wanted to select patients who demonstrated the worst and best clinical responses to either surgery, as determined by change in Lysholm scores between preoperative and 12-month postoperative scores. Furthermore, the number of samples that could be included in these preliminary investigations was limited to those that (1) had sufficient volumes of SF, (2) were not too diluted (through the lavage procedure used to collect SF at our center^32^) so as to be loaded onto ProteoMiner beads for dynamic range compression, and (3) did not have any blood staining, as this has been demonstrated to alter the detection of SF proteins.^44^ Therefore, it is important that these results not be overinterpreted. We did not undertake any bioinformatic pathway or network analyses on these proteomic findings because of the low number of samples and again to avoid overinterpretation of the results. In addition, the markers identified in this preliminary proteomic study will need to be validated in larger independent osteotomy and microfracture cohorts.

One of the proteins identified in the proteomic analyses was 14-3-3 protein theta (YWHAQ). This protein was of particular interest for the prediction of microfracture outcome because it was undetectable (via LC-MS/MS or ELISA) in the SF of individuals who responded well to this procedure. The LC-MS/MS analysis also highlighted this protein as being detected in only the nonresponders to osteotomy. However, 2 of the 4 samples used for proteomic analyses demonstrated detectable concentrations of YWHAQ when assessed with ELISA. This may be attributable to the sensitivity of the ELISA, or perhaps this protein’s detection was masked because of other more abundant proteins in the proteomic analysis. We measured YWHAQ in the larger osteotomy patient cohort (n = 13), and this protein was not found to have predictive value between responders and nonresponders to osteotomy. Therefore, we focused on the potential of this protein as a predictive marker for microfracture outcome. YWHAQ concentration was a significant variable in the linear regression model of microfracture outcome, indicating that with increasing preoperative concentrations of YWHAQ, postoperative Lysholm score decreases. When the sample was divided into responders and nonresponders to microfracture, based on an MCID of 10 Lysholm points, there was no statistical difference in concentration between the response cohorts. It is notable that in the full cohort of microfracture patients (n = 19) assessed, representing a more diverse range of clinical response compared with those assessed in the proteomic analyses, none of the SF samples of the responders had detectable levels of YWHAQ, whereas 55% of nonresponders had detectable concentrations. Therefore, there is value in testing this candidate biomarker protein in a larger cohort of individuals treated with microfracture. YWHAQ is a member of the 14-3-3 protein family, which has a wide range of functions, largely related to signal transduction pathways.^15^ The role of this specific isoform is not clear, particularly in relation to its function in OA or cartilage tissue damage or repair. However, it has been identified in several other relevant proteomic studies that assessed (1) the membrane proteins of equine articular chondrocytes^23^; (2) the secretome of cultured chondrocytes^29^; (3) the SF of patients with either rheumatoid arthritis or OA^2^; and (4) the SF of rabbits subjected to ACL transection compared with sham injury.^21^ Together, these data suggest that this protein may be secreted from chondrocytes into the SF, but how changes in the abundance of this protein within the SF relate to OA disease severity or progression requires further investigation.

In this study, we have started to develop clinical prediction models for microfracture and osteotomy by performing multiple regression analyses. Specifically, we generated a linear regression model that can correlate baseline SF HA, SF YWHAQ, SF ADAMTS-4, SF LYVE-1, and plasma HA concentrations along with patient smoker status and sex with Lysholm score at 12 months after treatment with microfracture. Moreover, although a weak correlation, a GLM with elastic net penalization was also generated for microfracture outcome; this again highlighted SF HA concentration, SF ADAMTS-4 activity, and baseline Lysholm score as predictors of postmicrofracture Lysholm score. Furthermore, we generated a generalized linear regression model with elastic net penalization that highlighted the activity of ADAMTS-4 enzyme in SF alongside patient smoker status and age as promising predictors of osteotomy outcome. These markers and prediction models need to be assessed in larger, independent cohorts to confirm their clinical utility.

Both regression models of microfracture outcome highlighted that lower preoperative SF concentrations of HA are predictive of higher postoperative Lysholm score and, hence, better knee function and less pain after treatment with microfracture. HA is a nonsulfated glycosaminoglycan that forms an important component of articular cartilage and the synovial membrane, and is highly concentrated in the SF.^3^ Altered concentrations of HA within the SF have long been considered a marker of degenerative joint disease,^30^ and serum HA levels can predict the progression of knee OA.^37^ Notably, baseline levels of HA were higher in individuals whose OA had progressed compared with those with earlier stage OA.^37^ Hence, we suggest that in our study, individuals who demonstrated better knee function after microfracture may have earlier-stage OA (and lower concentrations of HA), contributing to their clinical success compared with others who had more progressive preoperative OA changes (higher levels of HA) and poorer surgical outcomes. The potential utility of this marker for highlighting, preoperatively, which individuals have more progressive OA is strengthened by the fact that although the preoperative Kellgren-Lawrence score was included as a variable in the statistical modeling, it did not significantly contribute to the model prediction value. Moreover, there was not a significant difference in the baseline Kellgren-Lawrence scores between the microfracture responder and nonresponder cohorts. Therefore, assessment of SF HA could perhaps be indicative of early OA severity level that is below the detectable level using radiography in this cohort. The immunoassay that we used in this study (Corgenix) is a sandwich protein binding assay that has microwells coated with a highly specific bovine HA–binding protein to capture HA and uses an enzyme-conjugated version of HA-binding protein as a secondary antibody to detect and measure HA in the samples. Higher and lower molecular weight HAs have different molecular properties, and both have been assessed for therapeutic and prognostic use in OA.^5,48^ Therefore, in future studies to investigate the potential of HA as a candidate predictive biomarker of microfracture outcome, it would be of interest to assess the contribution of each molecular weight variant of HA and the biological role this protein plays.

The regression models of osteotomy and microfracture outcome indicate that lower activity level of ADAMTS-4 in the SF preoperatively is a predictor of better patient-reported knee function after osteotomy or microfracture. This finding is akin to our previous work that demonstrated that the absence of detectable ADAMTS-4 in the SF was predictive of success after ACI.^45^ ADAMTS-4, also known as aggrecanase-1, is more active in the SF of individuals with OA.^20,49^ This enzyme cleaves large chondroitin sulphate HA–binding proteoglycans, including aggrecan, a key structure of articular cartilage.^34^ Loss of aggrecan is a key driver in the progression of OA.^4,35,40^ Therefore, increased or detectible activity of ADAMTS-4 in SF preoperatively may indicate that the joints of these patient have OA that has progressed to a stage where realignment of the joint through treatment with osteotomy or stimulation of innate repair via microfracture surgery is insufficient to delay or halt the progression of their OA, explaining the propensity for their treatment to fail. Although the model indicated that ADAMTS-4 activity is indicative of osteotomy outcome, when patients were separated into responders and nonresponders to this surgery based on an MCID of 10 Lysholm points, there was no significant difference in ADAMTS-4 activity between the response cohorts. We suggest that when patients were separated into osteotomy responder and nonresponder categories, the difference in the activity of the ADAMTS-4 enzyme was not statistically significant because of the low numbers of patients within each arm, particularly as none of the patients who were classified as clinical responders had detectable enzyme activity, whereas activity levels were detectable in 3 of the 5 nonresponders. The same situation could also have affected the HA levels recorded. Since the initiation of this study, patients treated with osteotomy at our center have not routinely completed Lysholm scores preoperatively or at 12 months after surgery, thereby deeming it difficult to increase the study numbers. We accept that this is a limitation and therefore highlight the importance of independent validation.

In summary, we have generated a linear regression model that can be used to help predict Lysholm score after treatment with microfracture surgery. This model has highlighted a number of candidate SF and plasma biomarkers alongside patient demographic characteristics that have the potential to predict microfracture outcome. Further, the activity levels of ADAMTS-4 in SF, alongside patient smoker status and age, have the potential to predict the outcome after osteotomy. These protein markers and patient demographic characteristics carry the possibility of identifying individuals who are likely to demonstrate functional improvement after these surgeries such that the most appropriate surgical interventions can be offered to these patients, preventing the burden of treatment failure and the need to reoperate or provide an additional treatment. Alternatively, further research aimed at understanding the biological processes underlying the altered abundance of these proteins in individuals who respond poorly to these surgical interventions could provide novel therapeutic targets for the personalized augmentation of these surgeries: for example, administering aggrecanase inhibitors coincidently alongside osteotomy surgery. These candidate predictive biomarkers need to be further validated in larger independent cohorts; however, this work provides a foundation for the identification of biomarkers to predict outcome to interventions aimed at delaying or halting the progression of OA.

## Supplemental Material

sj-pdf-1-ajs-10.1177_0363546521995565 – Supplemental material for Identification of Candidate Synovial Fluid Biomarkers for the Prediction of Patient Outcome After Microfracture or Osteotomy Supplemental material, sj-pdf-1-ajs-10.1177_0363546521995565 for Identification of Candidate Synovial Fluid Biomarkers for the Prediction of Patient Outcome After Microfracture or Osteotomy by Charlotte H. Hulme, Mandy J. Peffers, Gabriel Mateus Bernardo Harrington, Emma Wilson, Jade Perry, Sally Roberts, Pete Gallacher, Paul Jermin and Karina T. Wright in The American Journal of Sports Medicine
