# Supplementary material for: Identification of Candidate Synovial Fluid Biomarkers for the Prediction of Patient Outcome After Microfracture or Osteotomy
Source: Am J Sports Med. 2021 Mar 31;49(6):1512–23. doi: 10.1177/0363546521995565 (PMC13021000; doi:10.1177/0363546521995565)
Supplement: sj-pdf-1-ajs-10.1177_0363546521995565 – Supplemental material for Identification of Candidate Synovial Fluid Biomarkers for the Prediction of Patient Outcome After Microfracture or Osteotomy [file sj-pdf-1-ajs-10.1177_0363546521995565.pdf]

A

	LC-MS/MS		ELISA	
	Fold-change	ANOVA (p-value)	Fold-change	t-test (p-value)
**YWHAQ**	infinity	0.0003	Infinity	0.0005
**LYLVE-1**	5.98	0.03	3.82	0.01

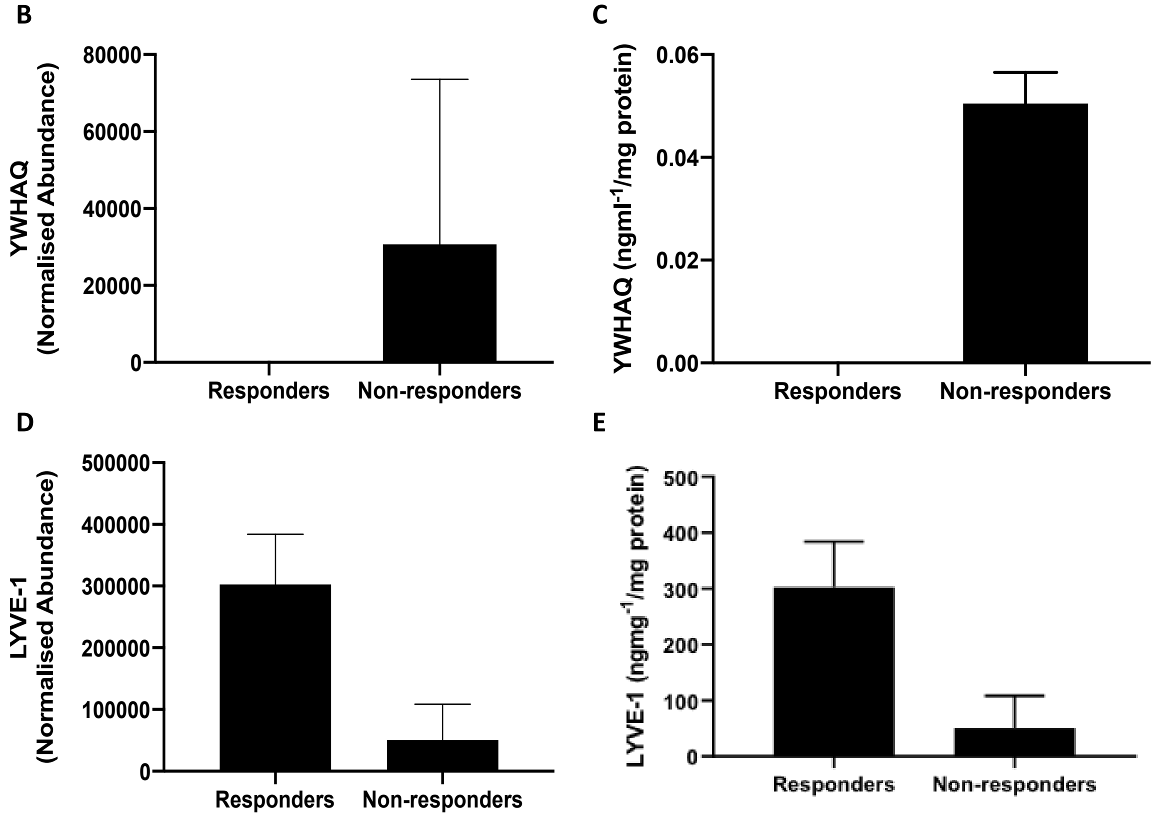

Append Figure A1. Two proteins, 14-3-3 protein theta (YWHAQ) and Lymphatic Vessel Endothelial Hyaluronan Receptor-1 (LYVE-1), that were identified by proteomic analysis as differentially abundant in the SF of non-responders compared to responders of microfracture were validated by ELISA. (A) Table demonstrating fold changes and p-values of differences between responders (n=3) cf. non- responders (n=3) to microfracture. YWHAQ was detectable in the SF in non-responders (n=3) to microfracture but not in the SF of responders (n=3) as demonstrated by (B) label-free mass-spectrometry and (C) validated using quantitative ELISA. LYVE-1 was increased in abundance in the SF of responders (n=3) cf. non-responders (n=3) to microfracture as demonstrated using B) label-free mass-spectrometry and (C) validated using quantitative ELISA. Data are mean±SD.

Append Table A1: A generalized linear regression model with elastic net penalization for Predictors of the Post-Microfracture Lysholm Score

Microfracture (n=19)			
**Component**	**R^2 Value**	**RMSE**	**Variable Importance**
Total Model	.519	26.4	
HA ($mgml^{-1}$)			100
Baseline Lysholm			28
ADAMTS-4 ($ngml^{-1}$)			15

Final elastic net model parameters were alpha- 1, lambda- 7.82. Alpha is a value between 0 and 1, where 0 is pure ridge regression, 1 is pure LASSO and values between are a mixture of both. Lambda is the shrinkage factor applied to model coefficients

*Abbreviations: ADAMTS-4 (A disintegrin and metalloproteinase with thrombospondin motifs 4), HA (hyaluronan), RMSE (*Root mean square error)
